# Supplementary material for: Distinct higher-order representations of natural sounds in human and ferret auditory cortex
Source: eLife. 2021 Nov 18;10:e65566. doi: 10.7554/eLife.65566 (PMC8601661; doi:10.7554/eLife.65566)
Supplement: Transparent reporting form [file elife-65566-transrepform1.docx]

***eLife’s* transparent reporting form**

We encourage authors to provide detailed information *within their submission* to facilitate the interpretation and replication of experiments. Authors can upload supporting documentation to indicate the use of appropriate reporting guidelines for health-related research (see [EQUATOR Network](http://www.equator-network.org/%20)), life science research (see the [BioSharing Information Resource](https://biosharing.org/)), or the [ARRIVE guidelines](http://www.plosbiology.org/article/info:doi/10.1371/journal.pbio.1000412) for reporting work involving animal research. Where applicable, authors should refer to any relevant reporting standards documents in this form.

If you have any questions, please consult our Journal Policies and/or contact us: [editorial@elifesciences.org](mailto:editorial@elifesciences.org).

**Sample-size estimation**

- You should state whether an appropriate sample size was computed when the study was being designed
- You should state the statistical method of sample size computation and any required assumptions
- If no explicit power analysis was used, you should describe how you decided what sample (replicate) size (number) to use

Please outline where this information can be found within the submission (e.g., sections or figure legends), or explain why this information doesn’t apply to your submission:

For the first experiment, we used 200 (40 * 5 models) different sounds, each repeated 4 times. This number of repetitions is lower than what is typically done for this type of study, but we gain statistical power from the high number of sounds. We had to limit the total number of sounds presented (800) to respect the maximal daily length of experimentation on an animal (each recording session lasted around 6hrs).

In the second experiment, we had less sounds (120, 60*2 models) but also used 4 repetitions to match with the first experiment, allowing a more direct comparison through the same pipeline.
(see Methods, Stimuli for Experiment I & II, Procedure for presenting stimuli sections)

**Replicates**

- You should report how often each experiment was performed
- You should include a definition of biological versus technical replication
- The data obtained should be provided and sufficient information should be provided to indicate the number of independent biological and/or technical replicates
- If you encountered any outliers, you should describe how these were handled
- Criteria for exclusion/inclusion of data should be clearly stated
- High-throughput sequence data should be uploaded before submission, with a private link for reviewers provided (these are available from both GEO and ArrayExpress)

Please outline where this information can be found within the submission (e.g., sections or figure legends), or explain why this information doesn’t apply to your submission:

Three ferrets were included in this study. For experiment I, we imaged auditory cortices on both sides for two ferrets (A and T). For experiment II, we imaged auditory cortices on both sides for one ferret (T), and only the left hemisphere for two ferrets (A and C). For ferret A, the right hemisphere was imaged with a different set of sounds (lacking speech and music sounds) and thus excluded from the study for simplicity. We note here that we checked that on the sounds that were common, the results were qualitatively similar to what is presented in this paper. For ferret C, only the left hemisphere was imaged for health-related reasons.
Each hemisphere is composed of a varying number of recording sessions (corresponding to different coronal planes) across days. Continuity of patterns across different slices could thus not be accounted for by a common noise as these are acquired separately (see Methods, Ultrasound imaging section).

**Statistical reporting**

- Statistical analysis methods should be described and justified
- Raw data should be presented in figures whenever informative to do so (typically when N per group is less than 10)
- For each experiment, you should identify the statistical tests used, exact values of N, definitions of center, methods of multiple test correction, and dispersion and precision measures (e.g., mean, median, SD, SEM, confidence intervals; and, for the major substantive results, a measure of effect size (e.g., Pearson's r, Cohen's d)
- Report exact p-values wherever possible alongside the summary statistics and 95% confidence intervals. These should be reported for all key questions and not only when the p-value is less than 0.05.

Please outline where this information can be found within the submission (e.g., sections or figure legends), or explain why this information doesn’t apply to your submission:

p-values and statistical testing methods are indicated in the text, alongside the concerned result and the reference to the figure. Dispersion and precision measures are described in the legend of figures.

(For large datasets, or papers with a very large number of statistical tests, you may upload a single table file with tests, Ns, etc., with reference to sections in the manuscript.)

**Group allocation**

- Indicate how samples were allocated into experimental groups (in the case of clinical studies, please specify allocation to treatment method); if randomization was used, please also state if restricted randomization was applied
- Indicate if masking was used during group allocation, data collection and/or data analysis

Please outline where this information can be found within the submission (e.g., sections or figure legends), or explain why this information doesn’t apply to your submission:

N/A

**Additional data files (“source data”)**

- We encourage you to upload relevant additional data files, such as numerical data that are represented as a graph in a figure, or as a summary table
- Where provided, these should be in the most useful format, and they can be uploaded as “Source data” files linked to a main figure or table
- Include model definition files including the full list of parameters used
- Include code used for data analysis (e.g., R, MatLab)
- Avoid stating that data files are “available upon request”

Please indicate the figures or tables for which source data files have been provided:

Our data is publicly available on Zenodo at the following link: <https://doi.org/10.5281/zenodo.5493682>

We provide ferret fUS data, before and after denoising, as well as additional files necessary to run our analyses.

Source code for our denoising procedure and production of main figures is available on <https://github.com/agneslandemard/naturalsounds_analysis>
